# Supplementary material for: Patterns and predictors of adherence to follow-up health guidance invitations in a general health check-up program in Japan: A cohort study with an employer-sponsored insurer database
Source: PLoS One. 2023 May 25;18(5):e0286317. doi: 10.1371/journal.pone.0286317 (PMC10212164; doi:10.1371/journal.pone.0286317)

**S1 Figure:** The interval between health check-ups in fiscal years 2017 and 2018 among adults who participated in both years


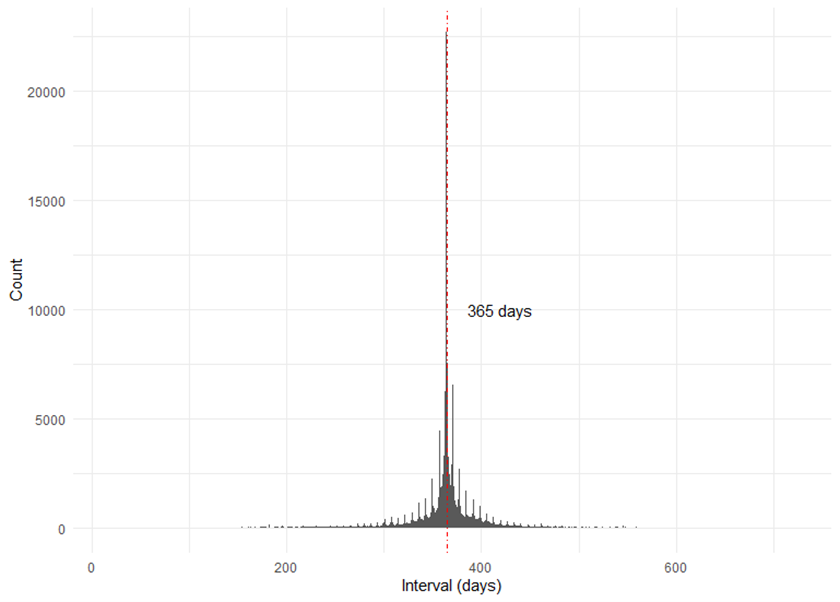

Supplement: S1 Fig — (DOCX) [file pone.0286317.s001.docx]
