# Supplementary material for: Patterns and predictors of adherence to follow-up health guidance invitations in a general health check-up program in Japan: A cohort study with an employer-sponsored insurer database
Source: PLoS One. 2023 May 25;18(5):e0286317. doi: 10.1371/journal.pone.0286317 (PMC10212164; doi:10.1371/journal.pone.0286317)

**S2 Figure:** Days between a health check-up in the fiscal year 2017 and health guidance for the first time since then among participants without prescribed medications for hypertension, diabetes mellitus, or dyslipidemia
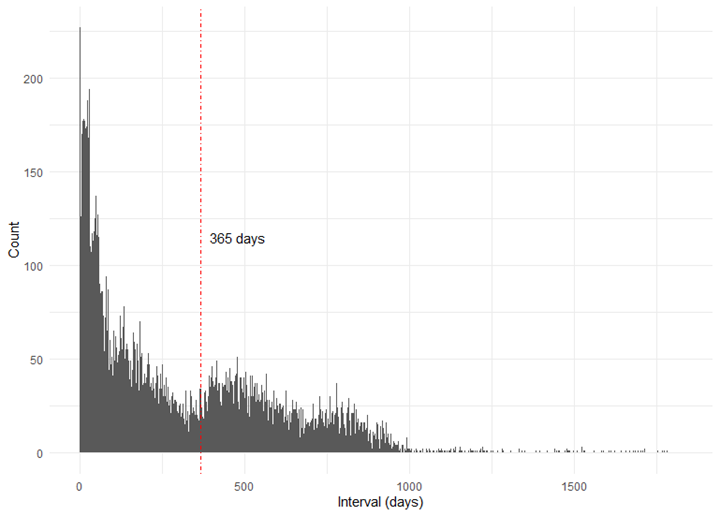

Supplement: S2 Fig — (DOCX) [file pone.0286317.s002.docx]
