# Supplementary material for: Patterns and predictors of adherence to follow-up health guidance invitations in a general health check-up program in Japan: A cohort study with an employer-sponsored insurer database
Source: PLoS One. 2023 May 25;18(5):e0286317. doi: 10.1371/journal.pone.0286317 (PMC10212164; doi:10.1371/journal.pone.0286317)
Supplement: Attachment — Submitted filename: Response to Reviewers (1).docx [file pone.0286317.s005.docx]

**Responses to Reviewers**

**Patterns and predictors of adherence to follow-up health guidance invitations in a general health check-up program in Japan: a cohort study with an employer-sponsored insurer database**

Our responses are provided in **bold** font, along with comments from editors and reviewers in *italics* font.

1. *They have partly discussed the limitations of the high CVD screening strategy used, but Comment 3 has not been specifically addressed in the discussion. Suggest add the following to the discussion as limitations of the screening strategy – (1) Elevated LDL was not considered a high-risk indicator; (2) People with low or normal BMI were excluded, and many of them may have had other CVD risk factors such as HT, DM, DL.*

**We appreciate the comment which clarifies the limitation of our manuscript. We modified the limitation paragraph reflecting the suggestion.**

**"... Third, the definition of the high CVD risk in Japan’s health check-up program, which emphasizes waist circumference, significantly differs from other commonly used risk prediction tools. For example, elevated low density lipoprotein cholesterol was not considered a high-risk indicator; and people with low or normal BMI with normal waist circumflex were excluded, though many of them may have had other CVD risk factors such as HT, DM, and dyslipidemia. This difference may limit the generalizability of our findings to individuals identified as high-risk using other risk prediction tools. …"**

2. *Line 147 - suggest “ .. over 400,000…” instead of “… 400 thousand …”*

**We appreciate the suggestion that enhances the readability of our manuscript. We edited the line accordingly.**

**“… We selected this insurer for the analysis because it is one of the largest employer-sponsored health insurers in Japan, covering over 400,000 individuals. In addition, the wording and design for the health guidance invitation remained consistent throughout the study period. …” (Data source paragraph in Methods)**
